# Predicting the growth suitability of *Larix principis-rupprechtii* Mayr based on site index under different climatic scenarios

**DOI:** 10.3389/fpls.2023.1097688

**Published:** 2023-02-02

**Authors:** Ruiming Cheng, Jing Zhang, Xinyue Wang, Zhaoxuan Ge, Zhidong Zhang

**Affiliations:** College of Forestry, Hebei Agricultural University, Baoding, China

**Keywords:** *Larix principis-rupprechtii*, random forest model, climate scenario, site index, growth suitability

## Abstract

*Larix principis-rupprechtii* Mayr (larch) is one of the main afforestation and timber production species used in North China. Climate change has led to a change in its suitable distribution and growth. However, the impact of climate change on its growth suitability is not clear. In this study, using forest resource inventory data and spatially continuous environmental factor data (temperature, precipitation, topography, and soil) in Hebei and Shanxi Provinces, China, the random forest model (RF) was used to simulate the larch site index (SI) and growth suitability under three shared socioeconomic pathways (SSPs: SSP1-2.6, SSP2-4.5, and SSP5-8.5) for the current and future (2021–2040, 2041–2060 and 2080–2100). The results revealed that (1) RF had excellent performance in predicting the regional SI (R^2^ = 0.73, MAE = 0.93 m, RMSE = 1.35 m); (2) the main factors affecting the productivity of larch were the mean temperature of the warmest quarter (BIO10), elevation (ELEV), mean diurnal range (BIO2), and annual precipitation (BIO12); and (3) larch currently had a higher SI in the Bashang areas and in the high-altitude mountains. The areas characterized as unsuitable, poorly suitable, moderately suitable, and highly suitable accounted for 15.45%, 42.12%, 31.94%, and 10.49% of the total area, respectively. (4) Future climate warming had an obvious inhibitory effect on the SI, and the effect strengthened with increasing radiation intensity and year. (5) The moderately suitable and highly suitable areas of larch growth showed a downward trend under future climate scenarios. By the end of this century, the suitable growth areas would decrease by 14.14% under SSP1-2.6, 15.17% under SSP2-4.5, and 19.35% under SSP5-8.5. The results revealed the impact of climate change on larch growth suitability, which can provide a scientific basis for larch forest management.

## 1 Introduction

Forest ecosystems play a central role in the global carbon cycle (Teets et al., 2018; Ameray et al., 2021), climate regulation (García-Valdés et al., 2020) and other ecological services (Kumar et al., 2008). Global climate change may have important impacts on forest productivity, biomass and phenological periods (Hof et al., 2021; Guo et al., 2022; Molina et al., 2022). Climate factors are important drivers of forest productivity, and future climate change may lead to a decline (Sabatia and Burkhart, 2014; Debaly et al., 2022) or an increase (Chen et al., 2020) in forest productivity. To assess future global climate change, the Sixth Assessment Report of the Intergovernmental Panel on Climate Change (IPCC AR6) developed an emission scenario combining a shared socioeconomic pathway (SSP) and a typical concentration pathway, and this method has been proven to be reasonable (Meinshausen et al., 2020). The change trends and impact mechanisms of forest productivity under different climatic conditions are currently unclear, which challenges forestry workers in formulating forestry strategies (Ogden and Innes, 2009). Therefore, under the background of continuous global warming, elucidating the change trend and influence mechanism of forest productivity has become a current research hotspot.

Accurate assessment of forest productivity is essential for sustainable forest management (Wang et al., 2015a; Socha et al., 2021; Achim et al., 2022). The site index (SI) is the most commonly used measure of forest productivity in forestry (Skovsgaard and Vanclay, 2008; Yue et al., 2016) and is usually defined as the dominant tree height or average dominant tree height of a stand at a given base age (Wang and Wang, 1994; Skovsgaard and Vanclay, 2008). Site-specific forest productivity is the result of a combination of biotic and abiotic factors (Sharma and Parton, 2018). The traditional SI calculations consider only the tree age factor (Sharma and Parton, 2019), and establish SI guidance curves or by compiling SI tables (Li and Zhang, 2010), which can accurately predict forest productivity at a small scale, but the process is very costly. For large spatial scales, forest productivity prediction accuracy tends to be low due to changes in site conditions and climatic factors (Zhu et al., 2019). Therefore, some studies (Bravo and Montero, 2001; Luis et al., 2003; Álvarez-Álvarez et al., 2011) used indirect methods to link variables such as topography, climate, and soil to the SI. Incorporating environmental variables into forest productivity prediction not only improved the model prediction accuracy but also increased the model dynamic prediction capability (Sharma and Parton, 2019). Sharma et al. (2015) simulated the SI of different tree species in northern Canada and predicted productivity changes under future climate scenarios; Antón-Fernández et al. (2016) assessed the SI of various tree species in Norway under the representative concentration pathway (RCP4.5) climate scenario; and Burkhart et al. (2018) simulated the future growth of *Pinus taeda* L. in the southeastern United States based on climate variables. Accordingly, in order to improve the predictive accuracy of productivity in different regions and forest types, we should consider different climate scenarios and key environmental factors.

To accurately assess forest productivity, various models have emerged, and the choice of different models may greatly affect the prediction results (Zhang et al., 2018). The generalized additive model (GAM) (Brandl et al., 2018), artificial neural network (ANN) (Aertsen et al., 2010), multiple linear regression (MLR) (Sharma et al., 2012), regression kriging (RK) (Watta et al., 2021) and random forest (RF) (Sabatia and Burkhart, 2014), etc., are widely used in forest productivity predictions. Among them, RF, proposed by Breiman (2001), is regarded as one of the most accurate nonparametric regression prediction methods. It shows good results in compensating for the shortcomings of parametric models (Aertsen et al., 2010; Cracknell and Reading, 2014), and exhibits lower sensitivity to different combinations of variables than other multivariable linear regression models (Hultquist et al., 2014; Liu et al., 2019). Because of the excellent prediction performance of RF, it has been widely used in studies such as larch-scale forest productivity prediction (Sabatia and Burkhart, 2014; Chirici et al., 2020; Horst-Heinen et al., 2021), biomass estimation (Ding et al., 2021), and the determination of the importance variables (Wang et al., 2021b).

*Larix principis-rupprechtii* (larch) is one of the main conifer species in North China and plays a key role in wood production, carbon sequestration, and ecological services (Di et al., 2014; Guo et al., 2022). It is generally distributed in pure and mixed forests at medium to high altitudes in alpine areas (Zhao et al., 2019), and alpine ecosystems have been shown to be more sensitive to climate change (Oddi et al., 2022). Research by Zhang et al. (2021) showed that future extreme drought conditions may cause larch growth to stagnate or even decline. The radial growth of *Larix gmelinii* (Rupr.) Kuzen. distributed in the Greater Khingan Mountains has shown a decreasing trend with increasing temperature (Jiang et al., 2016). Moreover, some studies have shown that the suitable distribution area of larch will have a tendency to migrate to high latitudes in the future (Mamet et al., 2019). The current research on larch productivity is based mostly on historical climate data (Li et al., 2021), but little is known about the potential productivity under future climate scenarios, and this lack of information is not conducive to the sustainable management of larch. Therefore, simulating the potential productivity and growth suitability of larch under current and future climate scenarios may provide a reference for improving our understanding of larch productivity and forest adaptive management in the context of climate change.

In this study, RF was used to predict the larch SI in the study area under current and future climate scenarios based on climatic, topographic and soil data. The specific objectives of this study were to (1) explore the main environmental factors affecting the larch SI; (2) simulate the larch SI under current and future climate scenarios in the study area; and (3) analyze the suitable growth areas of larch and the future change trend. Generally, temperature limits tree growth at high altitudes, whereas precipitation influences tree growth at low altitudes (Du et al., 2022). Currently, larch is mainly distributed in Bashang Plateau and mountains, and with future climate change, larch tree species would migrate to higher latitudes (Cheng et al., 2022). In the high-altitude suitable distribution area of larch, the effect of temperature on the larch growth may be more important than that of rainfall. Additionally, larch growth rate would tend to decrease with increasing time and emission scenarios (Wang et al., 2022). Therefore, we hypothesized that: (1) temperature has a greater effect on larch productivity than precipitation; and (2) larch productivity will be more inhibited by increasing years and radiation intensities.

## 2 Materials and methods

### 2.1 Study area

The study area is located in Hebei and Shanxi Provinces (110°14′-119°50′E, 34°34′-42°40′N), which is the native distribution area of larch in China. Hebei Province is located in the midlatitude coastal and inland intersection zone. The terrain is high in the northwest and low in the southeast. There are three major landform types, including the Bashang Plateau, Yan and Taihang Mountains, and Hebei Plain. Shanxi Province is located inland. It is a typical mountain plateau covered by loess. The study area is characterized by a temperate continental monsoon climate. The annual precipitation is 300-800 mm, and the annual temperature is 4-14°C. The soil types include brown soil, cinnamon soil, and chestnut calcareous soil. The dominant species include larch, *Pinus tabulaeformis* Carr., *Populus davidiana* Dode, *Betula platyphylla* Suk., and *Quercus mongolica* Fisch. ex Ledeb.

### 2.2 Data collection

#### 2.2.1 Site index data

**Figure 1:**
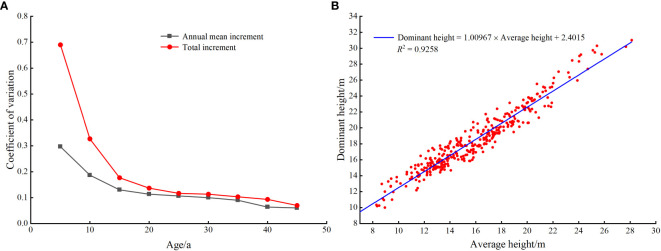
Coefficient of variation of dominant height growth **(A)**, and conversion equation of average height to dominant height **(B)** in larch plots.

**Figure 2:**
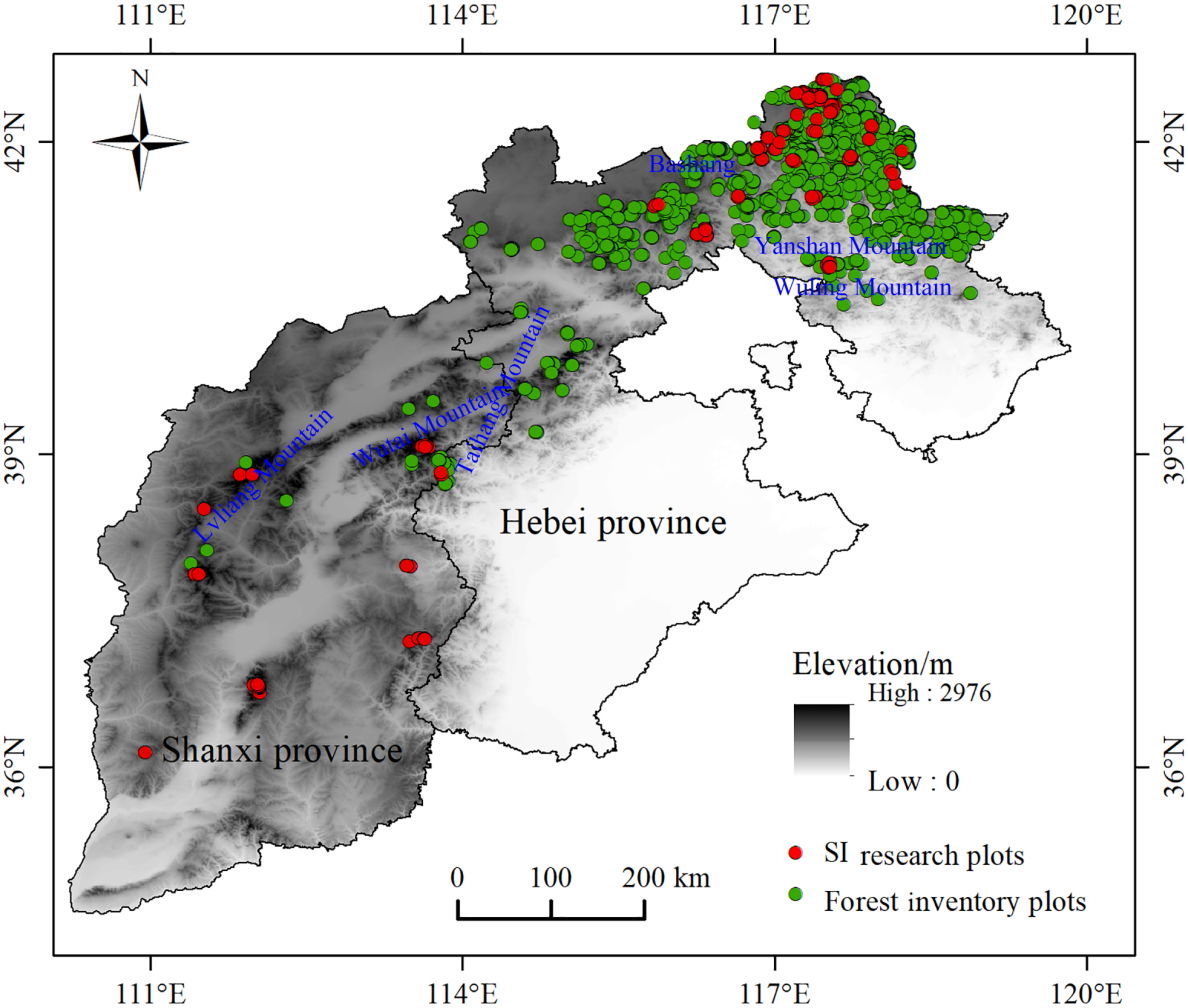
Study area and sample plot locations.

The actual larch SI was calculated based on the ninth national forest inventory data (2016-2020) in Hebei and Shanxi provinces, field survey data (temporary and permanent sampling plots) and destructive sampling data. Since most larch was located in nature reserves, we were unable to obtain tree-cutting permits for all sites. Therefore, using the research method and experimental data of Li et al. (2021), 20 larch plots with different site conditions were selected in the study area. Based on plot data, five dominant and five average trees were selected to identify destructive sampling trees in each plot. Finally, one dominant tree and one average tree representing the stand level in each plot were determined respectively, and then the destructive sampling data were obtained. The selection criteria for dominant trees included: good growth, no pests and diseases, the largest canopy, the thickest diameter at breast height, and the highest tree height. The selection criteria for average trees included good growth, no pests and diseases, and diameter at breast height and tree height equivalent to the stand mean values. The total tree height growth increment and average annual growth increment were calculated based on destructive sampling data. As shown in **Figure 1**, the dominant height growth gradually stabilized after 20 a, and the sample size was the largest at 30 a, so the baseline age was finally determined to be 30 a. From 2015 to 2021, sample plots ranging in size from 400 m^2^ to 900 m^2^ were set up in the study area. The average height of the stand and the average height of dominant trees were obtained (100 largest diameter at breast height trees per hectare). The sample plot data beyond ±3 times standard deviation was removed. We finally retained 337 plots to establish the mean stand height and dominant height transformation equation (*R* ^2^ = 0.9258) (**Figure 1B**). The transformation equation was: dominant height=1.00967×average height+2.4015. Using 30 years of larch plot data obtained from national forest inventory data, the average height of dominant trees was calculated by the transformation equation. We finally obtained the actual SI of 2576 plots (**Figure 2**, **Table 1**).

**Table 1:** Summary statistics of forest inventory data.

Items	Number of plots	Minimum	Maximum	Mean	Standard deviation
Average height/m	337	3.70	28.12	15.81	3.69
Dominant height/m	337	3.88	31.03	18.35	3.87
Site index/m	2576	5.53	22.10	9.67	2.59

#### 2.2.2 Environmental variables

The current and future (2021-2100) climate data with a 30 arc-second resolution were obtained from the WorldClim database (http://www.Worldclim.org) (Fick and Hijmans, 2017). Climate data adopted the SSPs developed by the IPCC AR6, including four core scenarios: SSP1-2.6, SSP2-4.5, SSP3-7.0 and SSP5-8.5 (Waliser et al., 2020; Petrie et al., 2021). We selected the medium-resolution Beijing Climate Center Climate System Model version 2 (BCC-CSM2-MR), which is widely used in China, to derive the future climate change scenario data. The SSP2-4.5 climate scenario is more consistent with future climate change trend in the study area (Lv et al., 2019a). SSP1-2.6, SSP3-7.0, SSP5-8.5 represent low, medium-to-high, and high emission scenario, respectively. The two climate scenarios SSP1-2.6 and SSP5-8.5 represent the two extremes. Therefore, three future climate scenarios, including low radiation intensity (SSP1-2.6), medium radiation intensity (SSP2-4.5) and high radiation intensity (SSP5-8.5), were used. We simulated the SI of larch species in the current and future three periods: 2030s (2021-2040), 2050s (2041-2060) and 2090s (2081-2100). Finally, 19 bioclimatic variables related to current and future temperature and precipitation were obtained. The digital elevation model (DEM) with a 30 arc-second resolution from the WorldClim database (http://www.Worldclim.org) was obtained, and ArcGIS 10.2 was used to generate elevation, slope, and aspect factors (ESRI Development Team, 2019). Soil data were obtained from the National Earth System Science Data Center shared platform (http://www.geodata.cn), and 12 soil data were selected: total nitrogen, total phosphorus, total kalium, available potassium, alkali-hydrolysis nitrogen, available phosphorus, bulk density, clay, powder grain, sand, rock fragment and organic matter. Soil data were resampled to raster data with a 30 arc-second resolution using ArcGIS 10.2.

Pearson correlation analysis and variance inflation factor (VIF) analysis were used to solve the collinearity of environmental factors (Radosavljevic et al., 2014; Jiang et al., 2018; Dang et al., 2021). Environmental factors with |r|<0.8 and VIF < 10 were selected. Finally, 19 environmental factors were retained (**Table 2**).

**Table 2:** Environmental factors used for building models.

Environment variables	Code	Variable name	Unit
**Temperature**	BIO2	Mean diurnal range (mean of monthly (max temp – min temp))	°C
	BIO4	Temperature seasonality (standard deviation × 100)	-
	BIO10	Mean temperature of warmest quarter	°C
**Precipitation**	BIO12	Annual precipitation	mm
	BIO17	Precipitation of warmest quarter	mm
**Terrain**	ELEV	Elevation	m
	SLP	Slope	°
	ASP	Aspect	%
**Soil**	AK	Soil available potassium	mg·kg^-1^
	AN	Soil alkali-hydrolysis nitrogen	mg·kg^-1^
	AP	Available phosphorus	mg·kg^-1^
	TK	Soil total kalium	g·kg^-1^
	TN	Soil total nitrogen	g·kg^-1^
	TP	Soil total phosphorus	g·kg^-1^
	SOM	Soil organic matter	%
	GRAV	Soil rock fragment	%
	CLAY	Percentage of clay in soil	%
	SAND	Percentage of sand in soil	%
	BD	Soil bulk density	g/cm^3^

### 2.3 Data analyses

#### 2.3.1 Random forest model

The RF model is an ensemble model that combines multiple decision trees (Breiman, 2001), which improves the accuracy and stability of the model. The final output is the average of all decision tree results. The “RandomForest” package was used in R 4.1.2 (R Core Team, 2022). RF contains three important model parameters: the number of features tried at each node (mtry), the number of trees (ntree), and the minimum node size. In this study, the number of features tried at each node (mtry) was set to the default value (1/3 of the total number of predicted variables), the minimum node size was selected according to the literature (Wang et al., 2015b), and 1000 value of ntree were constructed to ensure the stability of the results (Sabatia and Burkhart, 2014; Yang et al., 2016). Nineteen factors related to climate, topography and soil were included in the RF model for predicting larch SI distribution. 80% of the actual SI data were set as training data, and the remaining 20% were used as testing data. Three different criteria, including the coefficient of determination (*R* ^2^), root mean square error (RMSE), and mean absolute error (MAE) were used to evaluate the simulation accuracy (Castaño-Santamaría et al., 2019; Li et al., 2021).

#### 2.3.2 Importance of environmental factors

In this study, the importance function in the “randomForest” package in R 4.1.2 was used to calculate the average value of the mean decrease accuracy (%IncMSE) and mean decrease gini (IncNodePurity) for each environmental factor. “%IncMSE” is the percentage increase in the mean squared error, which is considered more important when the prediction error of the model increases for each randomly assigned variable. Thus, a larger value indicates a greater importance of the variable. However, IncNodePurity is measured by the residual sum of squares. The higher the node purity is, the more important the variable is. The larger the value is, the higher the importance of the variable (Angermueller et al., 2016; Niu et al., 2021). Due to the difference in the evaluation results of the importance of the two variables, the most important environmental factors affecting the SI were finally determined according to the consistency results of “%IncMSE” and “IncNodePurity”.

#### 2.3.3 Division of suitable growth areas for larch

RF was used to simulate the SI of larch under different climate scenarios. We imported the prediction results into ArcGIS 10.2 and drew maps of the suitable growth areas for larch. We used the raster calculator in ArcGIS 10.2 to normalize the SI results so that the raster values ranged from 0 to 1. The growth characteristics of larch were regrouped into four classes of suitable growth areas: unsuitable area (0.00-0.20), poorly suitable area (0.20-0.40), moderately suitable area (0.40-0.60), and highly suitable area (0.60-1.00) (Li et al., 2021). Moreover, we explored the change trend of the suitable areas for larch under future climate scenarios, and statistical analysis was carried out on the areas with different suitability values.

## 3 Results

### 3.1 Importance of environmental factors

**Figure 3:**
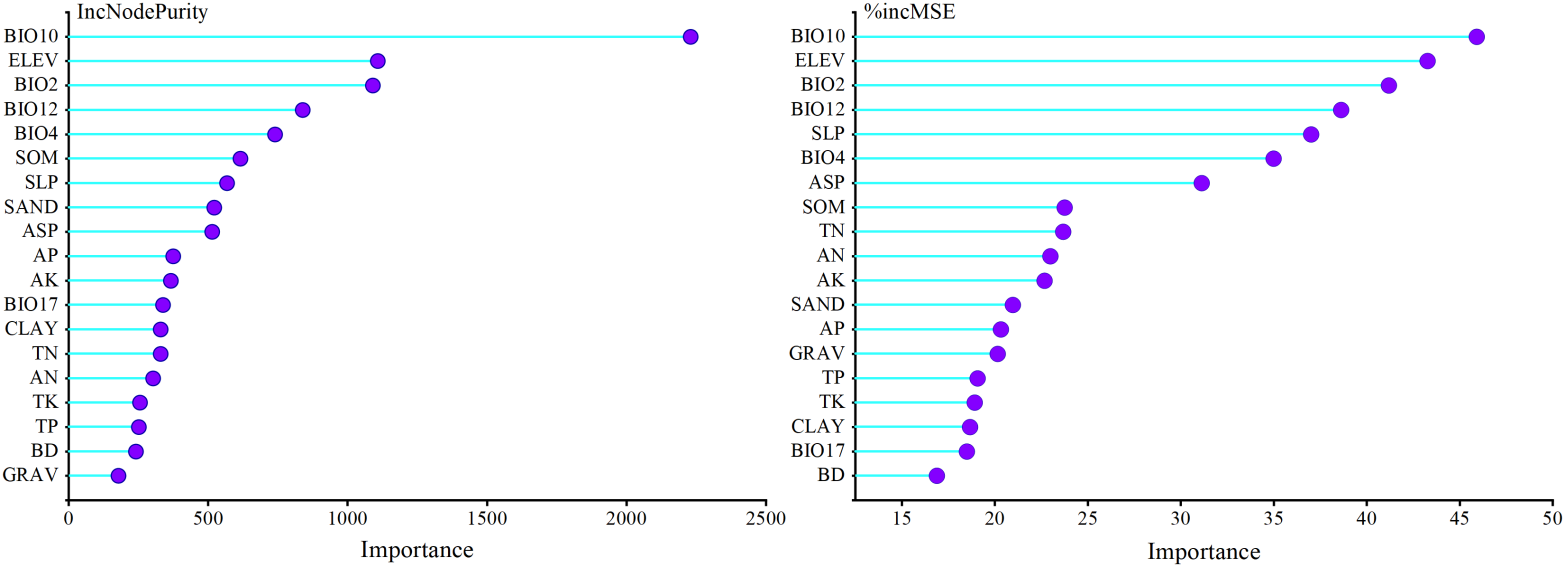
Importance of environmental factors for larch site index based on random forest results.

Environmental variable importance was ranked according to the “%IncMSE” and “IncNodePurity” values in the RF model (**Figure 3**). The consistency of the two results showed that the mean temperature of the warmest quarter (BIO10), elevation (ELEV), mean diurnal range (BIO2), and annual precipitation (BIO12) had the greatest impact on the larch SI. The total relative importance of the four environmental variables in the two evaluation indicators of “%IncMSE” and “IncNodePurity” were 41.55% and 59.17%, respectively. BIO10 (%IncMSE = 45.92%, IncNodePurity = 2229.93) and BIO12 (%IncMSE = 38.62%, IncNodePurity = 838.73) both had the most important impact on the SI. Among the soil variables, soil organic matter (SOM) had the greatest impact on the SI.

### 3.2 Model accuracy evaluation

**Figure 4:**
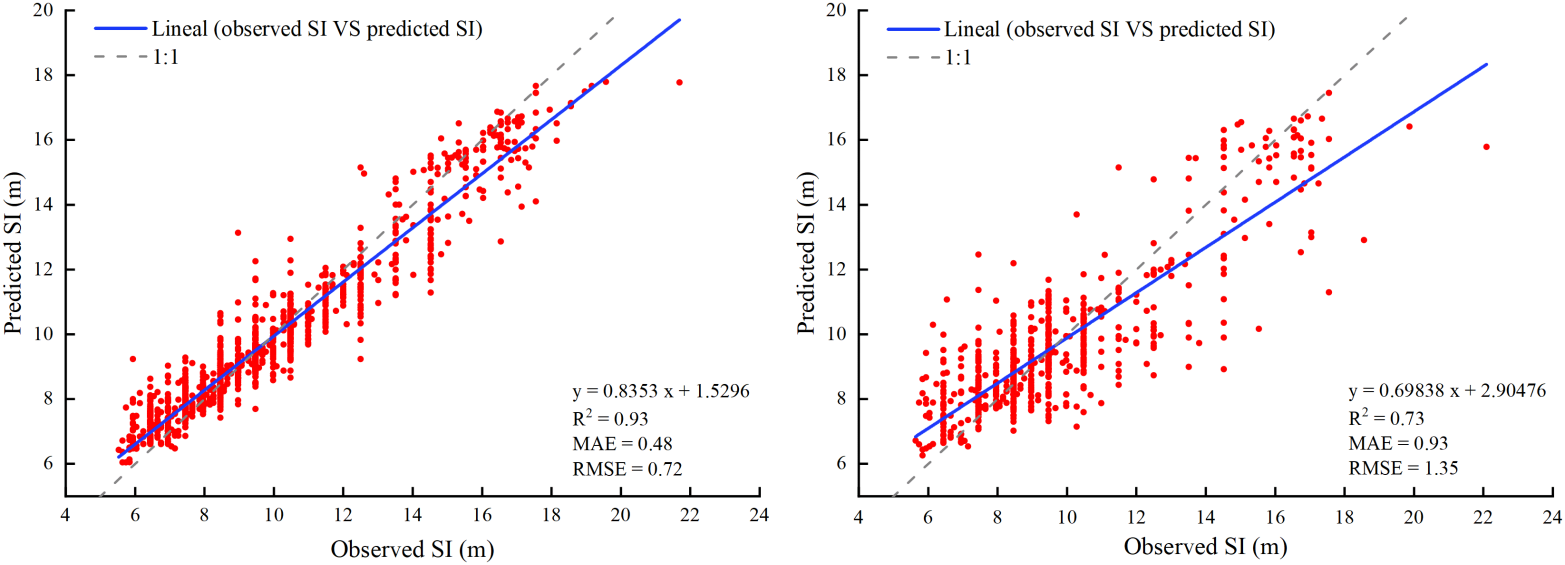
Comparison between observed and predicted values of SI for larch in training (left) and validation (right). Solid lines indicate the regression fitting results.

For training data, the *R* ^2^, MAE, and RMSE values were 0.93, 0.48 m, and 0.72 m, respectively. For test data, the *R* ^2^, MAE, and RMSE values were 0.73, 0.93 m, and 1.35 m, respectively (**Figure 4**). The results showed that the model fit was excellent and could accurately estimate the larch SI in the study area.

### 3.3 Current site index and growth suitability

**Figure 5:**
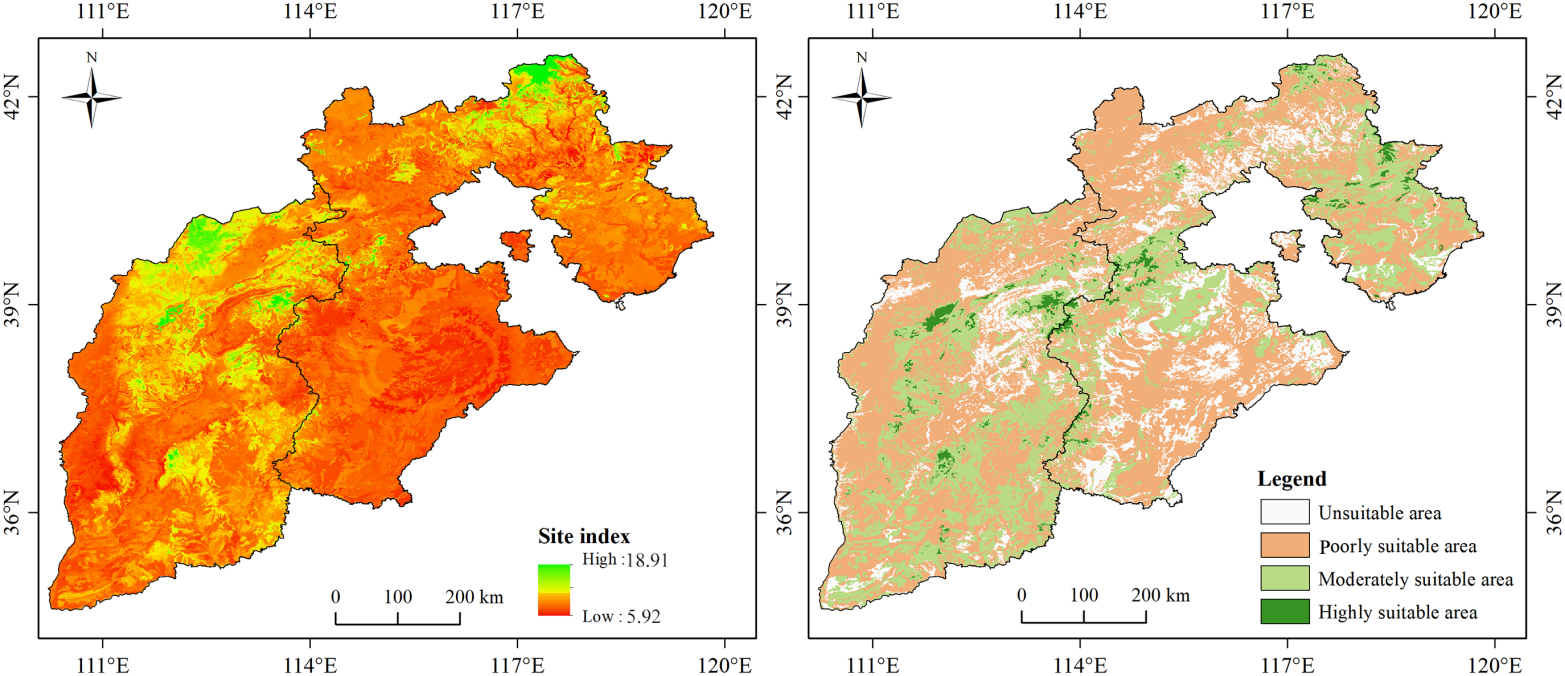
Spatial distribution pattern of larch SI (left) and suitable areas for larch growth (right) in current climate condition.

**Figure 6:**
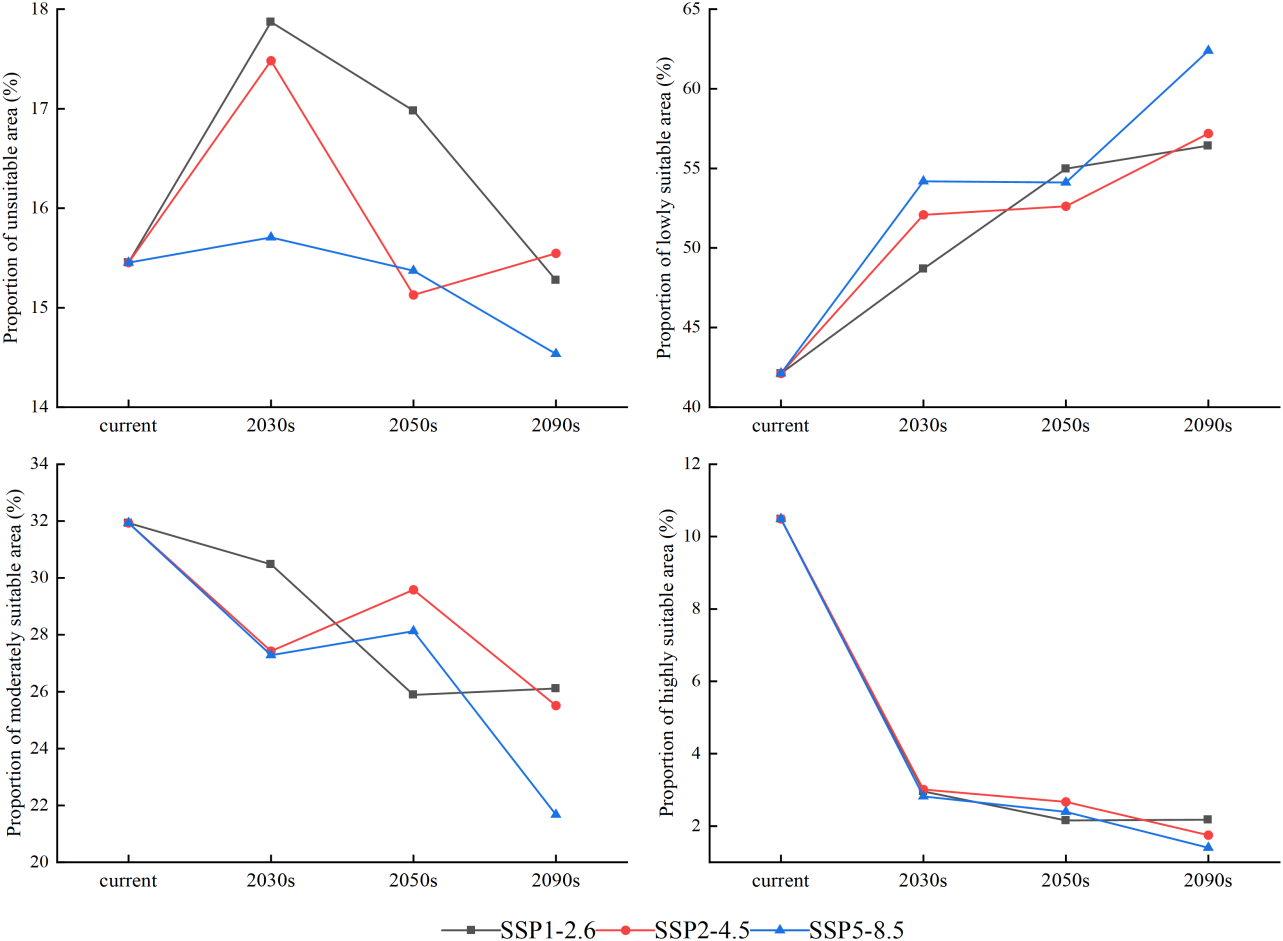
Area proportions of different suitable growth regions of larch over different periods (current, 2030s, 2050s, and 2090s) under SSP1-2.6, SSP2-4.5, and SSP5-8.5 climate scenarios.

Under the current climatic conditions, the actual SI of larch in the study area was in the range of 6.0-17.8 m, and the simulation results were in the range of 5.9-18.9 m (**Figure 5**). The simulation results were consistent with the actual values. The areas with a higher SI were mainly concentrated in the Bashang and high-altitude mountain areas, while scattered in the Yanshan Mountain, Taihang Mountain, and Lvliang Mountain. The SI was below mean in most areas of the North China Plain. According to the growth suitability classification of larch, the unsuitable area, poorly suitable area, moderately suitable area, and highly suitable area accounted for 15.45%, 42.12%, 31.94%, and 10.49%, respectively (**Figure 6**).

### 3.4 Site index and change trend under future climate scenarios

**Figure 7:**
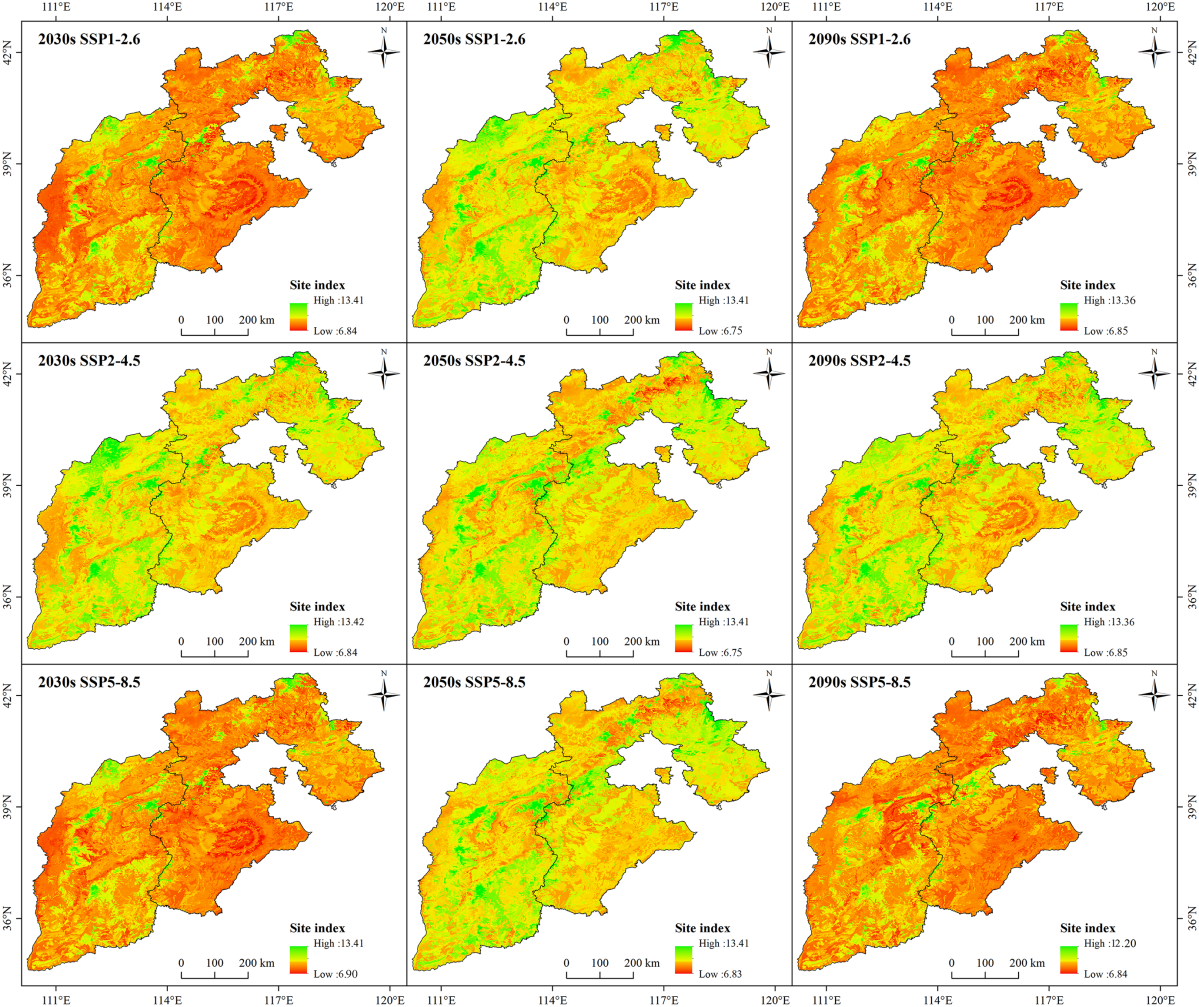
Spatial distribution patterns of larch SI under future climate scenarios (SSP1-2.6, SSP2-4.5, and SSP5-8.5).

The growth distribution areas changed significantly under the future climate scenarios (*p*<0.05) (**Figure 7**). The high-growth areas would be mainly distributed along the mountains. Additionally, there was a tendency for the high growth area to shrink over time. Among the scenarios, the SSP5-8.5 climate scenario had the most significant performance at the end of the century (2090s), and the SI values of larch in the study area ranged from 6.8 to 12.2 m, with a mean value of 8.4 m. The SI of larch was below 8.4 in most areas, accounting for 68.8% of the total area. However, the SI remained high (>8.4) only in the sporadic mountain tops, accounting for 31.2% of the total area.

**Figure 8:**
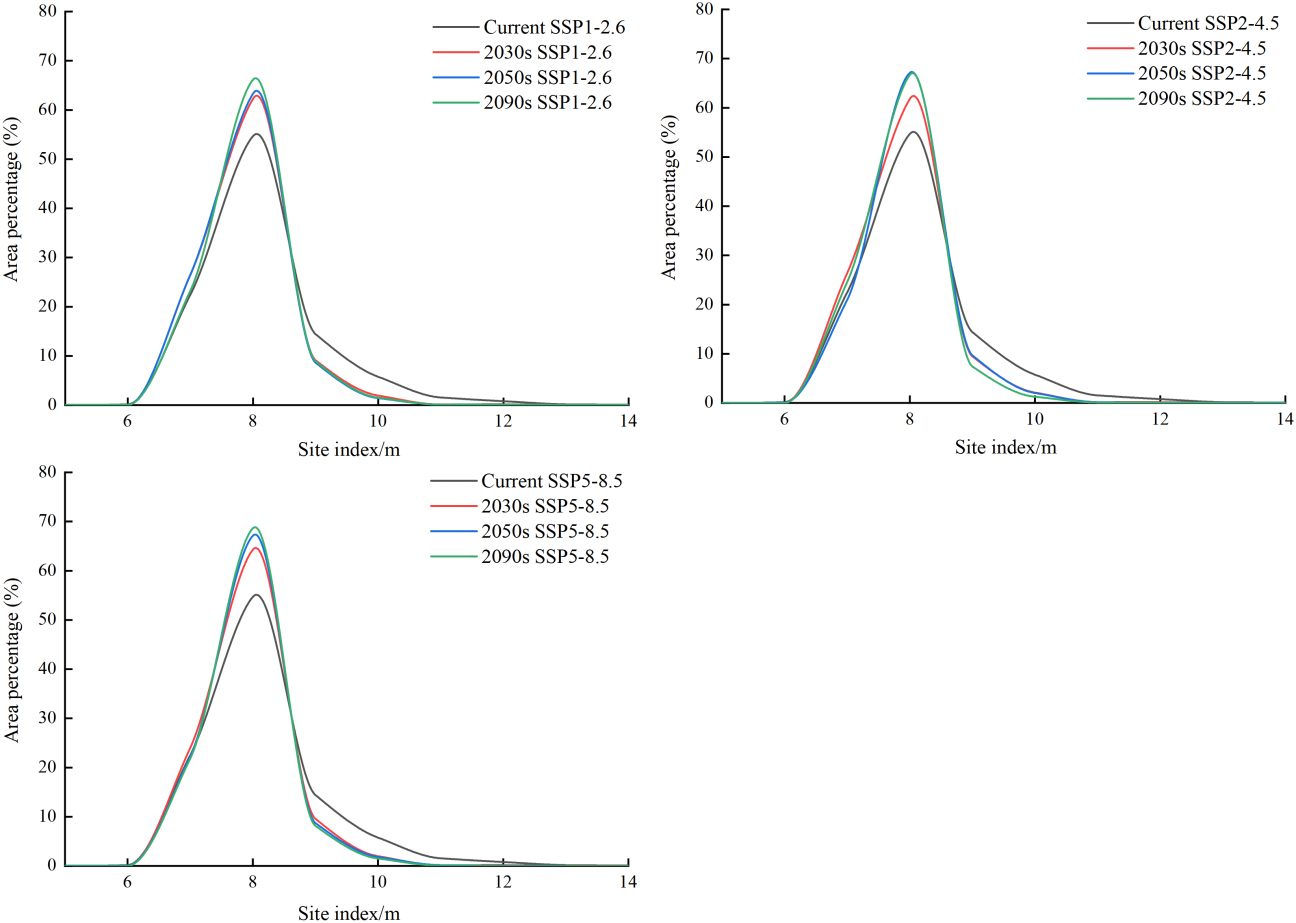
Area proportions of the distribution regions with different larch SI values for current and future periods (2030s, 2050s, and 2090s) under SSP1-2.6, SSP2-4.5, and SSP5-8 climate scenarios.

The potential SI distribution trend line was drawn to further understand the current and future potential SI changes for larch (**Figure 8**). Under each climate scenario, the area of the low growth distribution areas (SI<7) showed inapparent change, increasing peak for the moderate growth distribution areas (7<SI<9), whereas the area of the high growth distribution areas (SI>9) showed a decreasing trend from the current to the 2090s periods. With the increase in radiation intensity, the growth distribution areas with high SI decreased more sharply, and the change in the low value was not obvious.

### 3.5 Growth suitability distribution and area change trend under future climate scenarios

**Figure 9:**
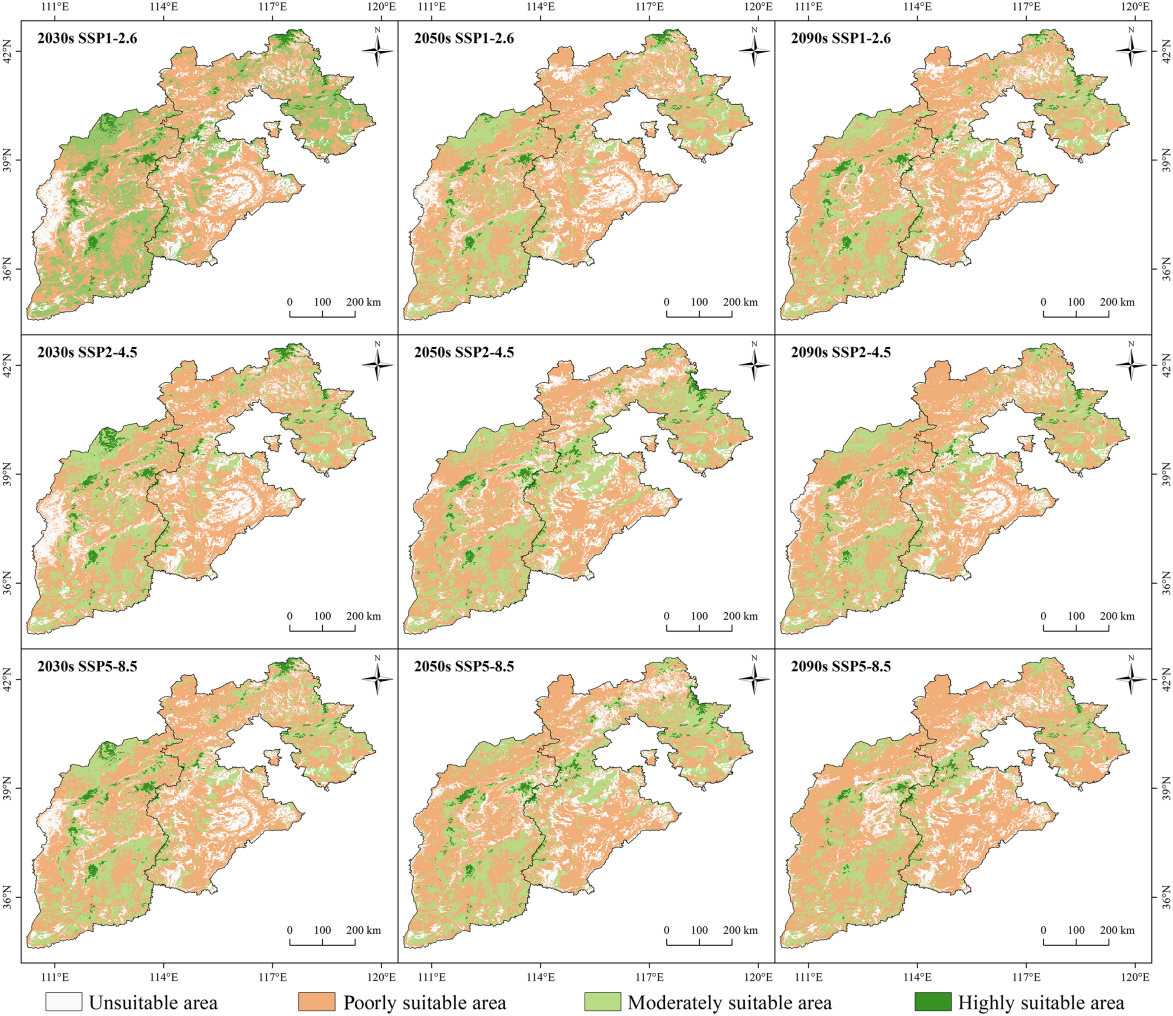
Spatial distribution patterns of larch suitable growth areas under different climate scenarios (SSP1-2.6, SSP2-4.5, and SSP5-8) in different future periods (2030s, 2050s, and 2090s).

The suitable areas showed different degrees of changes over time and with the increase in radiation intensity (**Figure 9**). In the same climate scenario over time, the highly and moderately suitable areas significantly decreased, and the poorly suitable areas and unsuitable areas showed an increasing trend (*p*<0.05). With the increase in radiation intensity, there was a shrinkage phenomenon in the highly suitable growth area, which was the most significant under the SSP5-8.5 climate scenario (*p*<0.05). In 2090, the highly suitable area of larch was scattered only in Wutai Mountain, Lvliang Mountain, Taihang Mountain, and Wuling Mountain. More than 80% of the study area would be transformed into unsuitable and poorly suitable areas.

The change rule of the unsuitable area was not obvious, while the poorly suitable area showed an increasing trend. The largest area increase occurred in the SSP5-8.5 climate scenario, from 42.12% to 62.39% until 2090 (**Figure 6**). Both highly and moderately suitable growth areas showed a downward trend. By the end of this century, the total moderately suitable area under the SSP1-2.6, SSP2-4.5 and SSP5-8.5 climate scenarios decreased by 5.82%, 6.42%, and 10.26%, respectively, and the highly suitable area was only 2.17%, 1.75%, and 1.40%, respectively.

### 3.6 Environmental factor response curve

**Figure 10:**
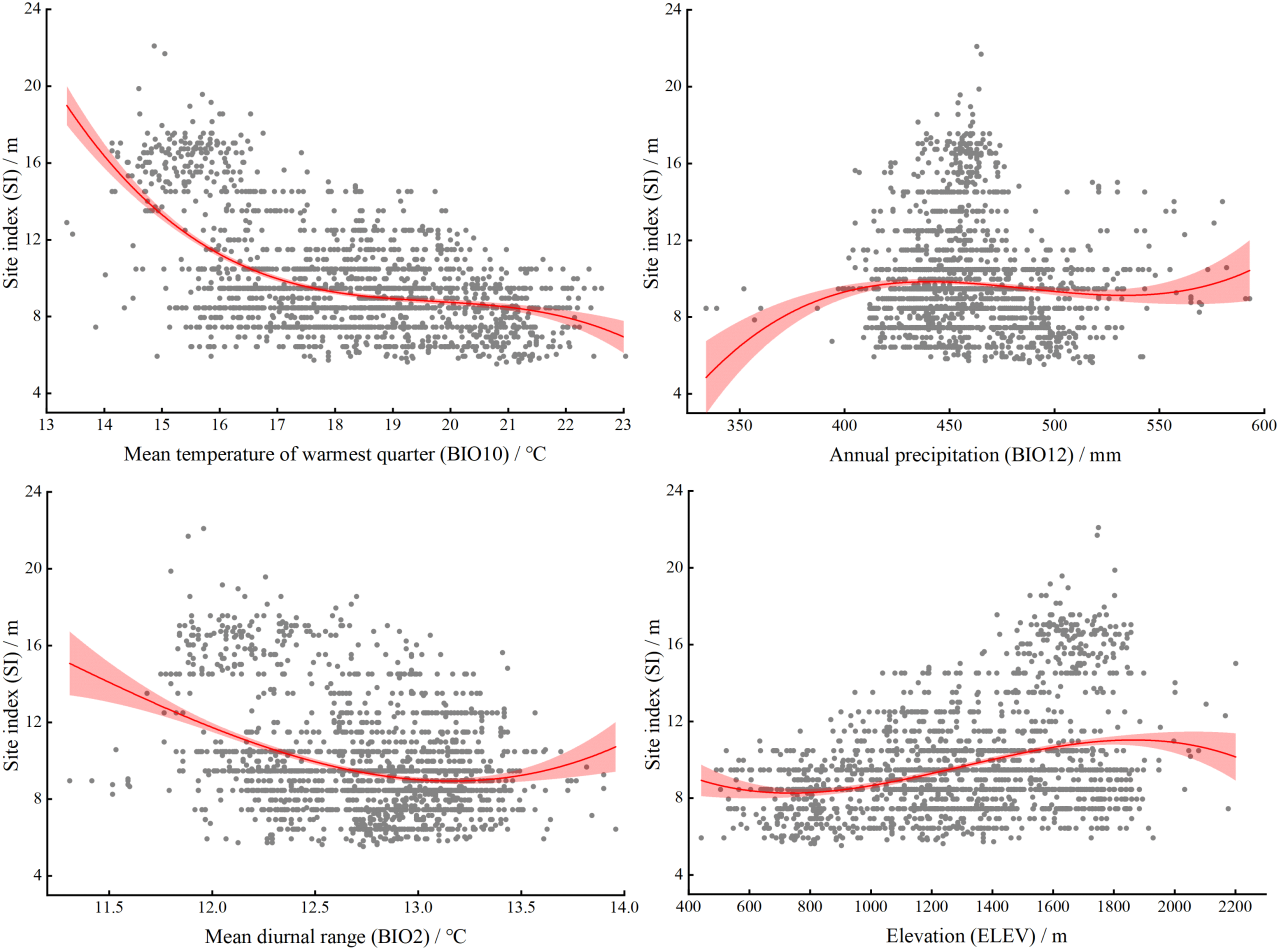
Response curves for the four factors included in larch SI model. The mean (red line) and standard deviation (light red area) of the probability presence. The prediction value of SI is shown as a function of each variable while all other variables are held at their median values at presence locations.

The potential SI of larch gradually decreased with increasing BIO10, and showed a stable stage between 17 and 21°C, and then showed a downward trend (**Figure 10**). The potential SI of larch showed an increasing trend with BIO12, and was in a stable stage between 400 and 550 mm, and then increased with increasing precipitation. The mean diurnal range (BIO2) in the study area was between 11 and 14°C, and the difference was small. The potential SI showed a significant downward trend with increasing BIO2 and then remained in a relatively stable state.

## 4 Discussion

In this study, based on a large number of sample plots, we obtained the actual SI through the mean stand height and dominant height transformation equation and achieved a good fitting effect (R^2^ = 0.9258) (**Figure 4**). However, compared with Li et al. (2021), who used the transformation equation to calculate the SI of larch plantations in northern Hebei Province, the equation fitting effect was poor. The possible reason for this difference was that the environmental differences caused by the large regional scale in this study led to the inconsistent growth of larch. Another reason may be that Li et al. considered only the larch plantation. Using the model to simulate the forest SI requires the model to explain at least 50% of the SI variation while satisfying the accessibility of auxiliary variables (Blyth and Macleod, 1981). We used RF to build a model based on the actual SI of the stand and environmental factor data. The model explained 73% of the SI variation and had a good prediction performance. Spatially explicit maps of larch SI constructed by RF model can help forest managers to clarify the distribution pattern of larch forest productivity and to develop specific management strategies.

Our study found that climate was the main factor affecting the productivity of larch in the study area. The results were highly consistent with other studies (Zang et al., 2016; Duan et al., 2022). The climatic factors affecting the site index of larch were mainly the temperature of the warmest quarter (BIO10), the mean diurnal range (BIO2), and the annual precipitation (BIO12) (**Figure 3**). BIO10 was the most important climatic factor affecting the SI of larch, which supported our first hypothesis. The study area has the highest temperature in summer, which is also the forest growing season. From the response curve, it was found that the growth of larch was not promoted with increasing temperature; in contrast, the growth of larch was inhibited with increasing temperature. Bowman et al. (2014) believed that suitable summer temperatures may improve forest productivity by promoting the photosynthesis of trees. However, when the temperature exceeds the optimum temperature for tree growth, high temperatures will lead to higher tree respiration and transpiration, resulting in a decline in forest productivity. It has also been suggested that high summer temperatures may increase drought stress, thereby restricting the radial growth of trees, especially in low and middle latitudes (Jiang et al., 2016). BIO2 reflects the temperature changes during one day, and the photosynthesis of plants during the day and respiration at night contribute to the accumulation of nutrients (Wang et al., 2021a). Some studies have shown that the diurnal temperature difference also affects the growth rate of plants (Pan et al., 2020). As the global climate continues to warm in the future, the limiting effect of temperature on larch growth may be further strengthened (Zhang et al., 2021). Precipitation has been proven to have a positive effect on the growth of larch (Shen et al., 2015; Lv et al., 2019a). In this study, the growth of larch tended to be stable when BIO12 was greater than 400 mm (**Figure 10**). Precipitation is an important restricting factor affecting tree growth, and it has a great impact on forest productivity in dry areas and a lower impact on areas with high rainfall (Luo et al., 2017). The average annual precipitation in most parts of the study area is more than 400 mm, so precipitation had little effect on the growth of larch. Xie et al. (2020) also showed that the increase or decrease in rainfall had little effect on the growth of larch.

Topographical factor, especially elevation, was also the important factor affecting larch productivity in the study area. The areas with a higher SI were mainly concentrated in the Bashang and high-altitude mountain areas, whereas the SI was low in low-altitude areas, for example, North China Plain. Temperature is the main climatic factor limiting the growth of trees at high altitudes, and the growth of low-altitude trees is mainly affected by precipitation (Du et al., 2022). As future temperature increases, the limiting effect of low temperature on tree growth is augmented at high altitude (Bai et al., 2019). At low altitudes, rapid evaporation of soil moisture may occur due to increasing temperature (Wu et al., 2021). Thus, trees at low elevations will be more vulnerable to drought stress, forcing them to migrate to higher elevations (Lv et al., 2019b). Our results indicate that under future climate scenarios, the highly and moderately suitable distribution areas of larch will be concentrated mainly in the high-elevation mountains due to increasing temperature and precipitation, while the low-elevation areas and plains areas will be transformed into poorly suitable and unsuitable area due to drought stress. Lv et al. (2019b) used a species distribution model to predict the suitable distribution areas of larch in Hebei province under future climate scenarios, and the conclusions also showed a trend of migration to higher elevation areas in the future, consistent with the results of this study. Larch can maintain a distribution of highly suitable area for growth in alpine areas, which is in line with its growth characteristics (Fang et al., 2019).

Under different climate scenarios in the future, the highly and moderately suitable areas of larch showed a downward trend, and with the increase in time and radiation intensity, the growth inhibition effect increased (**Figure 9**). Our finding supported the second hypothesis and was consistent with Wang et al. (2022) that the radial growth of larch distributed in northeast China will show a decreasing trend with increasing periods (from 2020s to 2080s) and increasing emission scenarios (from RCP2.6 to RCP8.5). Another similar study showed that larch productivity was higher under low-concentration climate scenario (RCP 2.6), and lower under high-concentration climate scenarios (RCP6.0 and RCP8.5) in the future (Shen et al., 2015). Increasing temperatures and decreasing rainfall during the growing season will cause frequent drought events in the future, which in turn will cause tree species to shift to north and higher elevations (Falk and Hempelmann, 2013). Our results verified this trend. This geographical shift resulted in a continuous decrease in the area of suitable habitats for larch from the SSP1-2.6 to the SSP5-8.5 climate scenario. The decline trend of larch productivity was consistent with the suitable distribution trend analyzed by the species distribution model under different climatic scenarios in the study area (Cheng et al., 2022). Therefore, we speculated that future climate warming may cause widespread mortality of larch in unsuitable and poorly suitable growth areas. Ashraf et al. (2013) proved the possibility of our speculation, and suggested that future climate warming will lead to higher winter temperatures, increasing the duration and frequency of winter melting and subsequent refreezing, ultimately leading to tree mortality. However, some studies suggest that future warming may promote larch productivity (Sato et al., 2016; Wu et al., 2021). Although many uncertainties remain, our study suggested that climate change, especially future climate warming, was an important factor influencing changes in larch suitable growth areas in Hebei and Shanxi regions, China.

Our study implied that varied forest management activities should be applied to maximize multifunctional benefits for larch forests across different suitable growth areas. In the moderately and highly suitable areas, increasing afforestation areas and developing large-diameter timber cultivation using larch trees are the priority, especially in Bashang areas and high-altitude mountains. Conversely, in unsuitable and poorly suitable areas, we should reduce larch afforestation areas and develop nature-based solutions to manage larch forests to maintain their basic soil and water conservation and other ecological functions. These specific forest management strategies are necessary to mitigate the adverse effects of climate change on larch forests. However, there were some limitations in the study. For example, we did not consider the impact of anthropogenic factors on larch productivity, which biased our results; in this study, we only considered the three most likely emission scenarios for future climate change, but did not include all climate scenarios. We should avoid these limitations in future studies to make the results more informative.

## 5 Conclusion

In this study, RF was used to evaluate the growth suitability of *L. principis-rupprechtii* in the study area under different future climate scenarios. The model had good prediction performance, and the results were highly reliable. The results showed that the suitable growth areas of *L. principis-rupprechtii* decreased significantly under different climate scenarios in the future. Under the SSP5-8.5 climate scenario, it was expected that by the end of this century (2090s), the unsuitable area, poorly suitable area, moderately suitable area, and highly suitable area would account for 14.54%, 62.39%, 21.67%, and 1.40%, respectively. Through the evaluation of the importance of environmental factors, it was concluded that temperature was the main driving factor affecting the potential SI of *L. principis-rupprechtii*, in which its growth was more stable when the temperature of the warmest quarter (BIO10) was between 17 and 21°C. The research results have guiding significance for the sustainable management and decision-making of *L. principis-rupprechtii*.

## Data availability statement

The original contributions presented in the study are included in the article/supplementary material. Further inquiries can be directed to the corresponding author.

## Author contributions

RC and JZ contributed to analysis and interpretation of data, and writing-original draft. XW and ZG performed acquisition and analysis of data, and writing-original draft. ZZ: conceiving the study and leading the writing. All authors contributed to the article and approved the submitted version.
